# Assessment of air pollution and its effects on the health status of the workers in beam rolling mills factory (Iran National Steel Industrial Group) from Ahvaz-Iran

**DOI:** 10.4103/0019-5278.50719

**Published:** 2009-04

**Authors:** Masoud Rafiei, Alaka S. Gadgil, Vikram. S. Ghole, Sharad D. Gore, Neemat Jaafarzadeh, Roksana Mirkazemi

**Affiliations:** Department of Environmental Sciences, University of Pune, Pune - 411 007, India; 1 Department of Geography, University of Pune, Pune - 411 007, India; 2 Department of Chemistry, Biochemistry Division, University of Pune, Pune - 411 007, India; 3 Department of Statistics, University of Pune, Pune - 411 007, India; 4 Department of Environmental Health Dept. Ahvaz Joundishapour University of Medical Sciences, Ahvaz, Iran; 5 Department of Ph.D student from Interdisciplinary School of Health Sciences, University of Pune, Pune - 411 007, India

**Keywords:** Beam and steel, relative risk, respiratory particulate matter

## Abstract

**Background:**

Air pollutants of iron- and steel-making operations have historically been an environmental and health hazard. These pollutants include gaseous substances such as sulfur oxide, nitrogen dioxide, and carbon monoxide. The Iran National Steel Industrial Group beam rolling mills factory has two production lines viz. line 630 and line 650, with different beam production capabilities and is capable of producing different types of beams.

**Materials and Methods:**

A retrospective cross-sectional study on 400 workers in different exposure levels to environmental pollution was performed during 2005 to determine the mean value of respirable particulate matter (RPM) concentrations and its effects on the health status of workers. To elicit information regarding the health status of the worker, the National Institute for Occupational Safety and Health standard questionnaire was used. Fisher's exact test was performed to assess the relative risk (RR) of exposure to air pollution on cardiovascular diseases, chest tightness, cough, difficulty in retention, i.e. loss of memory, tension, occupational fatigue, and occupational stress in exposed workers.

**Results:**

There was significant difference in RPM pollution level between two product lines. The RR of exposure to air pollution on cardiovascular diseases, chest tightness, cough, difficulty in retention, i.e. loss of memory, tension, occupational fatigue, and occupational stress in exposed workers were 2.78, 2.44, 2.15, 1.92, 1.57, 3.90, and 2.09, respectively.

## INTRODUCTION

Iron and steel factory workers are at risk of exposure to a wide range of pollutants. This depends on the particular process, the materials involved, and the effectiveness of monitoring and control measures. Adverse effects are determined by the physical state, propensities of the pollutant involved, the intensity and duration of the exposure, the extent of pollutant accumulation in the body, and the sensitivity of the individual to its effects. Some effects are immediate while others may take years and even decades to develop. Changes in processes and equipments along with improvement in measures to keep exposures below toxic levels have shown to be effective in reduceding the risks to the workers.[1]

The Iran National Steel Industrial Group (INSIG) is one of the biggest melting and casting plants in Iran. Consumption of raw material, energy, and water is high resulting in a significant volume of emission of air pollutants, both indoors and outdoors. Potential health effects depend on the number of particles in the respirable range, the chemical composition of the dust, the concentration of the exposure, and the duration of the exposure.[2] The main objective of the study was to determine the mean value of respirable particulate matter (RPM) concentrations in production line 650 and line 630 and to assess the relative risk (RR) of this pollution on the health status of exposed and non-exposed worker groups. The Beam Rolling Mills Factory has two production lines: line 630 and line 650, with the capacities of 190,000 and 195,000 ton annually, respectively, and is capable of producing various types of beams.[3]

## MATERIALS AND METHODS

A retrospective cohort study was conducted among workers of the INSIG. Two hundred workers (who were exposed to RPM at the work place) were randomly selected as the exposed group and 200 administrative staff were randomly selected as the non-exposed, control group. Exposed and non-exposed groups were preliminarily matched on various criteria such as age, work experience, and smoking habits. All 400 workers selected were asked to fill up the questionnaires. Data were analyzed using the 13^th^ version of the SPSS software, (Linda Fidder and *et al*., California State University, Bakersfield). Fisher's exact test was performed to calculate the RR.

To measure the air pollution, 40 sampling stations were selected in two different production lines. A low-volume sampler's pump was used for the study. After proper calibration and getting the calibration curve, data for RPM were collected (0.178 + 0.768X). The Model SKC (SKC Company, Blandford Forum, Dorset, United Kingdom), with a flow rate 2 L/min, membrane filter of 27 mm diameter, and pore size of 0.5 μm was used. Forty samples with the number of replications four times (160) in production lines 650 and 630 were collected. The concentrations were determined by weighing method using 0.1 mg precision balances.[5,6]

## RESULTS

There was significant difference in RPM pollution level between two product lines 650 versus 360. Table 1 shows that the mean concentrations of RPM in production line 650 were significantly different from the National Institute for Occupational Safety and Health standard (3 mg/m^3^), *P* < 0.05 (t-test).

**Table 1:** Comparison mean value of RPM* concentrations of separation in Lines 630 & 650 with NIOSH standard for 8 hours in terms of mg/m^3^

Source	Sample size	Number of sampling	Mean	Standard deviation	*P* Value	95% CI	

						Upper	Lower
630	320	20	3.09	± 1.43	0.76	2.56	3.82
650	320	20	3.78	± 1.03	0.03	3.48	5.04

* Respiratory particulate matter, NIOSH std. = 3 mg/m^3^, Statistical Method. T- Test

There were significant differences between work condition of the exposed and the non-exposed workers in neatness (*P* = 0.001), lighting (*P* = 0.001), and noise (*P* = 0.001) in the work place. Ninety-eight percent (*n* = 196) of the exposed workers reported suffering from dirty environment, 77% (*n* = 154) from lack of suitable light, and 99% (*n* = 198) from noisy working environment [Table 2].

**Table 2:** Work condition of exposed and non exposed group

Index	Exposed group (n=200)	Non group exposed (n=200)	Total	*P* Value
Neatness				0.001
Clean	4	171	175	
Dirty	191	29	220	
Very dirty	5	0	5	
Lighting				0.001
Very dark	13	0	13	
Somewhat dark	141	38	179	
Suitable	46	162	208	
Noise				0.001
Very suitable	2	15	17	
Relatively suitable	6	116	122	
Somewhat satisfactory	15	48	63	
Not satisfactory	177	21	198	

Table 3 shows that the RR of exposure to air pollution on developing cardiovascular diseases, chest tightness, cough, difficulty in retention, i.e. loss of memory, tension, occupational fatigue, and occupational stress in exposed workers were 2.78, 2.44, 2.15, 1.92, 1.57, 3.90, and 2.09 times, respectively, more among the exposed workers versus the non-exposed workers. Maximum RR was observed in occupational fatigue (RR = 3.90) and minimum RR was observed in feeling of tension (RR = 1.57).

**Table 3:** Regression coefficient between records of workers Disease and Indoor Mean value of RPM Concentration in Production lines 630 and 650

Disease	Sig. (2-tail)	R
Cardiovascular Disease	0.02	0.40
Chest tightness	0.001	0.70
Cough	0.001	0.71
Difficulty in Remembering	0.001	0.71
Tension	0.001	0.68
Occupational Fatigue	0.001	0.58
Occupational Stress	0.001	0.59

Table 4 illustrates the regression coefficient between records of workers disease and indoor mean value of RPM concentration in production lines 630 and 650. There was maximum and minimum regression coefficient between mean value of RPM concentrations and cough, difficulty in remembering (70%), and cardiovascular diseases (40%), respectively.

**Table 4:** Relative risk amongst exposed and non exposed group

Index (n=total)	Exposed (n)	Non exposed (n-200)	*P* value	RR (Confidence Interval)
Cardiovascular disease	25 (200)	9(200)	0.004	2.78 (1.3-5)
Chest tightness	22(200)	9(200)	0.015	2.44 (1.15-5)
Cough	43(200)	20(200)	0.015	2.15 (1.31-3)
Difficulty in remembering	43(200)	20(200)	0.003	1.92 (1.22-3))
Tension	74 (270)	47(130)	0.003	1.57 (1.16-2)
Occupational fatigue	178(270)	22(130)	0.001	3.9 (2.64-5)
Occupational stress	165 (270)	35 (130)	0.001	2.09 (1.56-2)

## DISCUSSION

The findings of this study show the direct effect of indoor air pollution on the increased risk of cardiovascular diseases, chest tightness, cough, difficulty in retention, i.e. loss of memory, tension, occupational fatigue, and occupational stress. Unacceptable work condition (unhygienic, noisy, and dark work environment) may also contribute to an increased risk of these problems. Unsafe behavior like lack of respiratory mask use may be another contributor.[7] The results of this study reveal the need for improvement of the work condition for the well-being of the workers. Generating awareness among the workers to use safety equipments is also another preventive measure that needed to be undertaken. It is recommended that the equipment of all sampling workstations be house kept and corrected. Periodic medical inspection of workers needs to be incorporated as mandatory management programs. The management should take these issues seriously for the well-being of the workers.[8]

## References

[1] Kiely P, Yap D, Brou GB, Fraser D, Dong W (1997). A comparative study of Toronto's air quality and selected world cities. Presented at the 90th Air and Waste Management Association.

[2] Kazuro I, Shoichi M, Yoji M, Toru M (2005). Correlation between suspended particles in the environmental air and causes of disease among inhabitants: Cross-sectional studies using the vital statistics and air pollution data in Japan. Environ Res.

[3] Iran National Steel Industrial Group (INSIG) (2004). Brochure literature.

[4] Debra K (1993). Nims, Basic of Industrial Hygiene, NIOSH, Indoor Air Qulity (IAQ) Questionnaires. American Industrial Hygiene Association.

[5] (1997). NIOSH, Particulates not otherwise regulated respirable, NIOSH Manual of Analytical Methods (NMAM).

[6] Baldini CN, Barszczewski E, Bernhardt L (1996). Standard practice for measurement of metals in workplace atmosphere by atomic absorption spectrophotometry, water and environmental technology atmospheric analysis. Annual Book of ASTM Standards.

[7] Ulrich F, Tuch T, Manjarrez M, Wiedensohler A, Herbarth O (2005). Indoor and outdoor submicrometer particles: Exposure and epidemiologic relevance. Environ Res.

[8] Ionescu A, Candau Y (2007). Air pollutant emissions prediction by process modeling: Application in the iron and steel industry in the case of a re-heating furnace. Environ Modelling Software.

